# Supplementary material for: Changes in Physical Performance among Community-Dwelling Older Adults in Six Years
Source: Int J Environ Res Public Health. 2023 Apr 19;20(8):5579. doi: 10.3390/ijerph20085579 (PMC10138314; doi:10.3390/ijerph20085579)
Supplement: Supplementary file 1 [file ijerph-20-05579-s001.zip › ijerph-2318428-supplementary.pdf]

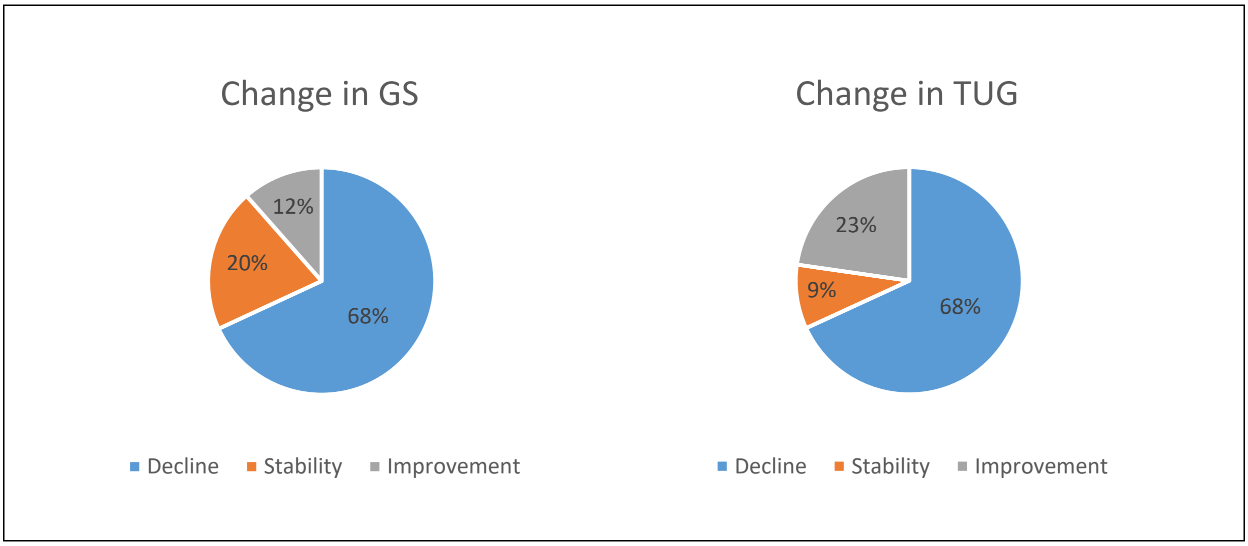

Figure S1: Change in physical performance tests - Gait Speed (GS) and Timed Up and Go (TUG) - from 2014 to 2019-20 of the older people in the COMO VAI? study.

Table S1: Mean Gait Speed (GS) in 2014 and 2019 according to sociodemographic, behavioral and health-related variables of the older adults in the COMO VAI? study. N=476. Pelotas, Brazil.

**Variables**	**GS in 2014**<br>*p-value*[a]<br>Mean (95%CI)	**GS in 2019**<br>*p-value*[a]<br>Mean (95%CI)
**Sex**	*p=0.001*	*p=0.025*
Male	1.16 (1.12; 1.21)	0.90 (0.85; 0.95)
Female	1.03 (0.99; 1.06)	0.83 (0.80; 0.8
**Age (completed years)**	*p<0.001*	*p<0.001*
60-69	1.13 (1.09; 1.16)	0.91 (0.88; 0.95)
70-79	1.03 (0.98; 1.08)	0.80 (0.75; 0.85)
80+	0.84 (0.74; 0.94)	0.60 (0.50; 0.70)
**Skin color**	*p=0.99*	*p=0.69*
White	1.08 (1.05; 1.11)	0.86 (0.83; 0.89)
Other than white	1.08 (1.01; 1.14)	0.84 (0.78; 0.91)
**Marital status**	*p=0.08*	*p=0.11*
Married/with a partner	1.10 (1.07; 1.14)	0.87 (0.83; 0.91)
Without a partner/separated	1.06 (0.99; 1.13)	0.79 (0.72; 0.85)
Widow(er)	1.03 (0.97; 1.08)	0.86 (0.81; 0.92)
**Economic level**[b]	*p<0.01*	*p=0.51*
A/B	1.14 (1.09; 1.19)	0.88 (0.83; 0.93)
C	1.05 (1.01; 1.08)	0.84 (0.80; 0.88)
D/E	1.02 (0.93; 1.11)	0.85 (0.76; 0.95)
**Education level (completed years)**	*p<0.001*	*p=0.016*
None	1.00 (0.91; 1.08)	0.78 (0.70; 0.87)
1-7	1.03 (0.99; 1.06)	0.83 (0.80; 0.87)
≥8	1.20 (1.14; 1.25)	0.92 (0.87; 0.97)
**Current work situation**	*p=0.26*	*p=0.30*
No (unemployed)	1.07 (1.04; 1.10)	0.85 (0.82; 0.88)
Yes (employed)	1.11 (1.05; 1;17)	0.88 (0.82; 0.94)
**Diet quality**[c]	*p=0.51*	*p=0.22*
Low	1.05 (1.00; 1.10)	0.82 (0.77; 0.87)
Average	1.09 (1.04; 1.13)	0.87 (0.82; 0.91)
High	1.09 (1.04; 1.14)	0.88 (0.83; 0.93)
**Leisure-time physical activity (>150 min/week)**[d]	*p=0.06*	*p=0.14*
No	1.06 (1.03; 1.09)	0.84 (0.81; 0.87)

Yes	1.13 (1.07; 1.19)	0.90 (0.84; 0.96)
**Smoking**	*p=0.37*	*p=0.78*
Not a smoker	1.06 (1.02; 1.10)	0.85 (0.81; 0.88)
Smoker	1.10 (1.02; 1.18)	0.86 (0.78; 0.93)
Former smoker	1.10 (1.05; 1.15)	0.87 (0.82; 0.92)
**Alcohol consumption**[e]	*p=0.027*	*p=0.79*
No	1.06 (1.03; 1.09)	0.85 (0.82; 0.88)
Yes	1.13 (1.08; 1.19)	0.86 (0.81; 0.92)
**Multimorbidity**	*p=0.37*	*p=0.20*
Up to 4 diseases	1.10 (1.05; 1.15)	0.88 (0.83; 0.93)
5 or more diseases	1.07 (1.03; 1.10)	0.84 (0.81; 0.88)
**Depression**[f]	*p=0.003*	*p=0.021*
No	1.09 (1.06; 1.12)	0.87 (0.84; 0.90)
Yes	0.97 (0.90; 1.05)	0.78 (0.70; 0.85)
**Polypharmacy**[g]	*p<0.001*	*p=0.026*
No	1.00 (0.95; 1.05)	0.81 (0.76; 0.86)
Yes	1.11 (1.08; 1.14)	0.88 (0.84; 0.91)
**Body Mass Index**[h]	*p=0.32*	*p=0.92*
Low weight	1.05 (0.94; 1.16)	0.87 (0.76; 0.98)
Normal	1.11 (1.06; 1.16)	0.86 (0.81; 0.91)
Overweight	1.06 (1.03; 1.10)	0.85 (0.82; 0.88)

[a] p value obtained through adjusted linear regression including variables according to hierarchical levels: 1st Level: sex, age, skin color, marital status, economic level, education, work status; 2nd Level: diet quality, physical activity, smoking, alcohol consumption; 3rd Level: multimorbidity, depression, polypharmacy and nutritional status.

[b] according to *Associação Brasileira de Empresas de Pesquisa* (ABEP)[21]: category A/B indicating higher socioeconomic status;

[c] assessed using the Diet Quality Index for the Elderly (*Índice de Qualidade da dieta do idoso* - IDQ-I)[22];

[d] assessed by the International Physical Activity Questionnaire (IPAQ)[23];

[e] alcohol consumption in the last month;

[f] according to the Geriatric Depressive Scale (GDS-10)[25,26];

[g] continuous use of five or more medications[27];

[h] cutoffs recommended by Lipschitz et al[28].

Table S2: Mean Timed Up and Go (TUG) in 2014 and 2019 according to sociodemographic, behavioral and health-related variables of the older adults in the COMO VAI? study. N=476. Pelotas, Brazil.

Variables	TUG in 2014 *p-value*[a] Mean (95%CI)	TUG in 2019 *p-value*[a] Mean (95%CI)
**Sample**	*Mean TUG 2014*	*Mean TUG 2019*
**Sex**	*p=0.018*	*p=0.21*
Male	9.24 (7.95; 10.52)	11.86 (10.57; 13.14)
Female	11.19 (10.25; 12.13)	12.90 (11.97; 13.84)
**Age (completed years)**	*p<0.001*	*p<0.001*
60-69	9.80 (8.86; 10.74)	10.78 (9.84; 11.73)
70-79	10.51 (9.22; 11.81)	13.14 (11.85; 14.44)
80+	16.13 (13.42; 18.85)	24.08 (21.36; 26.80)
**Skin color**	*p=0.40*	*p=0.55*
White	10.68 (9.85; 11.52)	12.48 (11.65; 13.32)
Other than white	9.83 (8.05; 11.62)	13.09 (11.31; 14.87)
**Marital status**	*p=0.13*	*p=0.42*
Married/with a partner	9.91 (8.91; 10.91)	12.21 (11.21; 13.20)
Without a partner/Separated	10.78 (8.83; 12.73)	13.64 (11.69; 15.59)
Widow(er)	11.86 (10.29; 13.43)	12.88 (11.32; 14.45)
**Economic level**[b]	*p=0.029*	*p=0.60*
A/B	11.85 (10.57; 13.13)	12.29 (11.01; 13.57)
C	9.68 (8.68; 10.67)	12.91 (11.91; 13.91)
D/E	9.19 (6.86; 11.52)	11.87 (9.54; 14.20)
**Education level (completed years)**	*p=0.09*	*p<0.001*
None	12.53 (10.31; 14.75)	16.40 (14.18; 18.62)
1-7	10.59 (9.60; 11.58)	12.82 (11.83; 13.80)
≥8	9.60 (8.27; 10.94)	10.61 (9.28; 11.95)
**Current work situation**	*p=0.71*	*p=0.25*
No (unemployed)	10.46 (9.58; 11.33)	12.75 (11.87; 13.62)
Yes (employed)	10.10 (8.47; 11.72)	11.64 (10.02; 13.27)
**Diet quality**[c]	*p=0.82*	*p=0.67*
Low	10.78 (9.43; 12.12)	12.98 (11.64; 14.33)
Average	10.22 (9.05; 11.39)	12.61 (11.35; 13.88)
Low	10.32 (9.04; 11.61)	12.14 (10.85; 13.43)
**Leisure-time physical activity (>150 min/week)**[d]	*p=0.63*	*p=0.039*

No	10.35 (9.54; 11.16)	13.02 (12.21; 13.83)
Yes	10.81 (9.15; 12.46)	11.07 (9.42; 12.73)
**Smoking**	*p=0.10*	*p=0.21*
Not a smoker	11.29 (10.26; 12.32)	13.22 (12.19; 14.25)
Smoker	9.63 (7.43; 11.82)	12.04 (9.85; 14.23)
Former smoker	9.56 (8.21; 10.91)	11.73 (10.38; 13.08)
**Alcohol consumption**[e]	*p=0.04*	*p=0.77*
No	10.05 (9.17; 10.93)	12.53 (11.65; 13.40)
Yes	11.93 (10.40; 13.46)	12.79 (11.26; 14.32)
**Multimorbidity**	*p=0.82*	*p=0.12*
Up to 4 diseases	10.55 (9.30; 11.80)	11.76 (10.51; 13.01)
5 or more diseases	10.36 (9.41; 11.31)	13.06 (12.11; 14.01)
**Depression**[f]	*p=0.059*	*p=0.041*
No	10.13 (9.36; 10.91)	12.28 (11.50; 13.05)
Yes	12.14 (10.21; 14.06)	14.44 (12.52; 16.37)
**Polipharmacy**[g]	*p=0.004*	*p=0.07*
No	12.23 (10.86; 13.59)	13.67 (12.30; 15.03)
Yes	9.77 (8.86; 10.68)	12.11 (11.21; 13.02)
**Body Mass Index**[h]	*p=0.023*	*p=0.43*
Low weight	14.81 (11.64; 17.99)	11.67 (8.50; 14.84)
Normal	10.29 (8.96; 11.62)	11.94 (10.61; 13.27)
Overweight	10.22 (9.27; 11.17)	12.93 (11.98; 13.88)

[a] p-value obtained through adjusted linear regression including variables according to hierarchical levels: 1st Level: sex, age, skin color, marital status, economic level, education, work status; 2nd Level: diet quality, physical activity, smoking, alcohol consumption; 3rd Level: multimorbidity, depression, polypharmacy and nutritional status.

[b] according to *Associação Brasileira de Empresas de Pesquisa* (ABEP)[21]: category A/B indicating higher socioeconomic status;

[c] assessed using the Diet Quality Index for the Elderly (*Índice de Qualidade da dieta do idoso* - IDQ-I)[22];

[d] assessed by the International Physical Activity Questionnaire (IPAQ)[23];

[e] alcohol consumption in the last month;

[f] according to the Geriatric Depressive Scale (GDS-10)[25,26];

[g] continuous use of five or more medications[27];

[h] cutoffs recommended by Lipschitz et al[28].
